# Artificial intelligence-based prediction for cancer-related outcomes in Africa: Status and potential refinements

**DOI:** 10.7189/jogh.12.03017

**Published:** 2022-04-23

**Authors:** John Adeoye, Abdulwarith Akinshipo, Peter Thomson, Yu-Xiong Su

**Affiliations:** 1 Division of Oral and Maxillofacial Surgery, Faculty of Dentistry, The University of Hong Kong, Hong Kong SAR, China; 2 Oral Cancer Research Theme, Faculty of Dentistry, The University of Hong Kong, Hong Kong SAR, China; 3 Department of Oral and Maxillofacial Pathology and Biology, Faculty of Dentistry, University of Lagos, Lagos, Nigeria; 4 College of Medicine and Dentistry, James Cook University, Cairns, Queensland, Australia

Globally, cancer ranks among the most common causes of death, especially among people under 70 years of age [1]. With this burden rapidly increasing globally, utilizing prediction tools to assist decision-making and encourage individualized treatment planning is gradually becoming paramount in cancer diagnosis and management. Notably, many tools constructed on the backend of artificial intelligence (AI) algorithms have been shown to improve the predictive accuracy and clinical impact of risk prediction compared to clinical scenarios not utilizing these models [2]. However, the actualisation of the potential of health care AI has mostly been assessed in high-income and resource-driven centres. The impact and efficiency of oncological AI-based prediction tools are expected to be better realised when applied in low-resource and rural settings fraught with a paucity of experienced clinicians and specialists.

Africa is considered the most vulnerable continent in the world with regards to the availability of resources for optimal health care provision and comprises about 85.2% of countries that belong to the low-income/lower-middle-income economies [3]. Also, many individuals have to rely on underfunded public health care systems, even for lethal diseases like cancer, which may be further constrained by access to secondary or tertiary health centers and financial limitations [4]. Therefore, AI prediction tools would prove beneficial in triaging patients before they present to health institutions, as well as for treatment selection of patients with precancer and cancer in such settings. Furthermore, these platforms could potentially be employed alongside other digitized systems such as telemedicine for remote consultations and Internet of Medical Things (IoMT) to improve referral strategies [5,6].

We examine the implementation status of AI platforms for cancer outcomes in Africa and provide pointers that could maximize their utilization.

## IMPLEMENTATION AND POTENTIAL REFINEMENTS FOR CANCER-BASED ARTIFICIAL INTELLIGENCE TOOLS IN AFRICA

**Figure 1:**
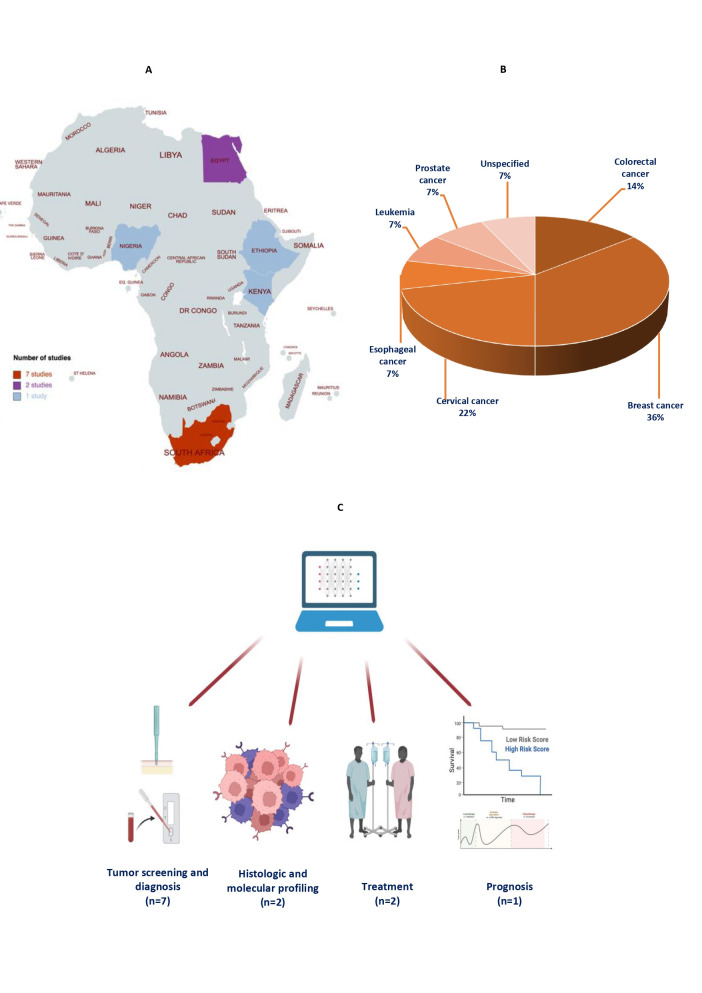
Oncological AI-based prediction models in Africa by a) Regions, b) Cancer types, and c) Modality of application.

The uptake of technology-driven systems available in developed regions is slower in Africa than in other parts of the world, as these platforms are often generated from integrative variables that may not be available for external validation and clinical application in many African settings. For oncology, this may be further challenged by the variation in molecular or etiological subtypes, as well as infrastructural and ethnocultural peculiarities of cancer management in the African region [7,8]. A preliminary search in five electronic databases to map tools developed from January 2000 to January 2022 using patient cohorts in Africa found 12 prediction tools for different malignancies and applications (Appendix S1 in the **Online Supplementary Document**). Their characteristics are presented in **Table 1** and **Figure 1**.

**Table 1:** Application and Implementation phases of oncological AI tools in Africa

Year of report	Type of AI tool	Predicted outcome(s)	Phase of implementation performed*	Method of validation performed	Accuracy / AUC	Reference
2021	Machine learning	Cell-free-DNA-based oesophageal cancer diagnosis	Phase I-III only	Internal	0.92	[9]
2021	Machine learning	Post-operative length of stay after colorectal cancer (CRC) resection	Phase I-III	Internal	0.82	[10]
2021	Deep learning	CRC recurrence and survival	Phase I-III	Internal	0.85	[11]
2021	Machine learning	Breast cancer tumour mutation burden	Phase I-III	Internal	0.74	[12]
2020	Natural language processing	Identification of malignant cases in electronic records	Phase I-III	Internal	0.93	[13]
2021	Machine learning	Breast cancer risk prediction	Phase I-III	Internal	0.98	[14]
2022	Natural language processing	Identification of demographic, clinical and molecular subtype information from records	Phase I-III	Internal	-	[15]
2020	Deep learning	Contouring of clinical treatment volumes and normal structures in cervical cancer radiotherapy	Phase III	External	0.94	[16]
2021	Natural language processing	Identification of malignant cases in electronic records	Phase I-III	Internal	0.92	[17]
2021	Deep learning	Detection of HSIL and LSIL in cervical cancer	Phase I-III	Internal	0.97	[18]
2018	Machine learning	Breast cancer staging	Phase I, II, IV	NIL	0.84	[19]
2021	Machine learning	Detection and typing of leukemic cells	Phase I-IV	Internal	0.98	[20]

AI – artificial intelligence, AUC – Area under the receiver operating characteristic curve, CRC – colorectal carcinoma

*Phase I – Data collection and processing, Phase II – Model construction, Phase III – Model validation, Phase IV – Software application development, Phase V – Impact and efficiency analysis, Phase VI – Model implementation in daily oncology practices [21].

Generally, the development of AI tools for assisted decision-making among patients with cancer in Africa can be considered a recent endeavour, with many models being reported from 2018 (**Table 1**). With more oncological AI platforms being proposed in the post-COVID-19 era, it may be that the refocusing of experienced health personnel towards pandemic control did motivate the search for automated tools aimed at facilitating the diagnosis, treatment selection, and prognostication of cancer patients [22-24]. Also, these tools may have been proposed with the aim of increasing the efficiency of specialists, easing the case burden caused by a worsening health provider-to-patient ratio [25], and limiting person-to-person contact to clinical encounters of utmost importance [26].

Relative to the different subregions in Africa, the pattern of AI penetrance mirrors the ongoing economic and infrastructural development in the continent [4,27]. As such, populations in areas with larger economies and fair technological expertise like South Africa, Egypt, Nigeria, and Kenya are largely the focus of the development and validation of the AI models for cancer outcomes (**Figure 1**, Panel A). This identifies a niche for intensified efforts in AI development and/or deployment among approximately 90% of the countries in Africa (especially within Central Africa), many of which are either low-income or lower-middle-income nations.

Moreover, oncological AI-based models in Africa were being considered for outcome determination among patients with breast, cervical, and colorectal carcinomas (**Figure 1**, Panel B). This fairly coincides with the incidence of malignant neoplasms in the continent, with breast, cervical, prostate, liver, and colorectal cancers being the five most common subtypes [1]. However, actionable AI patient-based platforms have not been proposed to facilitate the screening, diagnosis, and management of hepatocellular and prostatic carcinoma in the region. While one model was found for prostatic carcinoma [17], it was fashioned to improve incidence reporting in cancer registries from free-text pathology reports, which may not have a direct consequence on the diagnosis or management of the malignancy. Hence, Africa will benefit from research seeking to propose novel platforms and/or external validation of existing AI-based platforms, especially for liver and prostate cancer, as well as other, previously unconsidered malignancies [28-32]. Specifically, malignancies such as oesophageal cancer and Kaposi’s sarcoma, with higher age-standardized incidences in Africa than in other parts of the world, would markedly benefit from AI-based prediction to improve the standard and reach of diagnosis and management [1].

**Figure:**
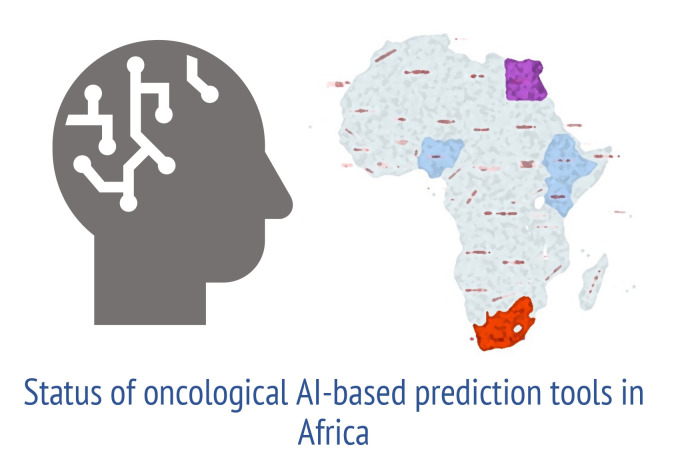
Photo: Oncological AI in Africa. Source: From the authors’ own collection, used with permission.

The technical aspects of the development and validation of cancer-related AI models in Africa is generally satisfactory (**Table 1**). This may be credited to the availability of knowledgeable individuals with sufficient skills to perform optimal data pre-processing, model selection, tuning, validation, and software deployment. However, many novel AI platforms that are being developed by African institutions affiliates may be employing open-source databases comprised of patients from other populations [33-37]. This alludes to the unavailability of clinical, imaging, pathologic, and molecular data from cancer patients, especially in electronic formats, within many parts of the regions. The data paucity challenge may have informed the modelling of many one-time point outcomes such as tumour diagnosis and profiling instead of prognosis, which requires time-to-event monitoring that is likely unavailable (**Figure 1**, Panel C). While this problem may be inseparable from the overall technological advancements and e-health penetrance in the continent [38], it is expected that an increase in the knowledge and potential impacts of digital health among African clinicians involved in cancer management, as well as health information technology stakeholders and policymakers, would encourage digitization of salient patient records and treatment follow-up data for model construction. Essentially, these knowledge provision avenues and platforms may also come in the form of practical experiences while participating in multinational collaborative work for external validation of prediction models. This strategy could potentially lay the foundation for the integration of these AI-based models with other digital health systems, especially telehealth [18]. In addition, the digital integrative framework of AI tools and telehealth can be adopted to mitigate the lack of direct access to experienced specialists and tertiary facilities, while delivering comparable health care standards in rural Africa. Training platforms may also emphasize the role of AI in assisting clinicians in their duties rather than replacing them, as the need to secure their jobs may discourage interest in digital health tools and their implementation [39].

The performances of the set of models proposed for use in African oncological settings so far were satisfactory to excellent upon validation [9-12,14,18] (**Table 1**). However, to fully actualize the potential of AI-based prediction for cancer outcomes in Africa, inquiries into the impact and efficiency of the models in comparison to the current standards of management are paramount. Since the training and validation of AI prediction platforms have only been described recently within the region, the future should see intensified efforts into elucidating their impact when implemented in practice. While this requires the use of prospective rigorous study approaches like the randomized controlled trial (RCT) design, findings of these pivotal research before full-scale implementation may take a few years. In the interim, Africa may benefit from studies seeking to perform an external validation of any AI-based prediction tools for cancer-related outcomes, especially when their clinical effectiveness is being investigated using an RCT. However, this will require an initial feasibility check to determine whether the input parameters of the model may be obtained within the region.

## CONCLUSION

Applying AI in developing credible tools for the prediction of cancer outcomes in Africa is a relatively recent endeavour. Model construction in the continent reflected the degree of technological advancement among the nations, as well as the availability of electronic health records and digitized clinical investigations. None of the models developed locally and internationally have been assessed for their impact and efficiency in automated assistive clinical decision-making and risk stratification. Hence, this should represent the phase of AI implementation to be addressed soon, especially in numerous remote areas of the low- and lower-middle-income areas. Likewise, for these settings, it is expected that the future will see the integration of AI-based prediction and other digital health technologies to mitigate the challenges of reduced manpower, substandard facilities, and lack of access to care currently experienced in cancer diagnosis and management in Africa.

## Additional material


Online Supplementary Document
