# Supplementary material for: Artificial intelligence-based prediction for cancer-related outcomes in Africa: Status and potential refinements
Source: J Glob Health. 2022 Apr 23;12:03017. doi: 10.7189/jogh.12.03017 (PMC9022723; doi:10.7189/jogh.12.03017)
Supplement: Online Supplementary Document [file jogh-12-03017-s001.pdf]

APPENDIX S1: MAPPING SEARCH

A comprehensive search was conducted to identify original studies published from January 1, 2000, to January 31, 2022, that focused on the construction and implementation of AI-based tools for cancer-related outcomes in any African setting. The composition of the African continent was according to the list of 54 countries and 4 territories available at https://www.worldometers.info/geography/how-many-countries-in-africa/. Furthermore, AI-based tools encompassed those developed on the backend of any machine learning (ML), deep learning (DL), or natural language processing (NLP) algorithms. Studies evaluating the use of other information technology systems such as IoMT, telehealth, virtual and augmented reality, and health ecosystems were selected if they also involved integration with an AI algorithm. We did not assess studies evaluating the perception or acceptance of health personnel or individuals to AI prediction in oncology or studies only highlighting data preprocessing methods for AI models even if they were in African settings. Articles written in English and French were sourced from five electronic databases – PUBMED, EMBASE, Web of Science, Cochrane Library, and Scopus. Search keywords comprised several texts of relevance to the review objectives which were focused using Boolean operators for individual databases (Table A1).

We adopted a dual-stage study selection exercise to identify important studies. Initially, the title and abstracts of the retrieved citations were screened to facilitate the selection of those that utilized AI algorithms and digital health

technologies in Africa for cancer-related outcomes. The full-length texts of studies screened successfully were then assessed according to the eligibility criteria for inclusion. Both selection processes were performed by two authors (JA and AA) independently and serial discussions were held for discordant selections. Agreement between both authors was the basis for final study selection. At this stage, a complementary manual search of the references and citations of selected studies was then performed to enable the inclusion of studies that may have been missed during electronic database searching.

We also abstracted data from individual studies in duplicate and independently using electronic spreadsheets. The full texts were referred to when different inputs were entered by these authors. Data items comprised the authors' names, publication year, location, study design, sample, type of malignancy, application modality and specific outcome, AI construction phase according to de Hond et al (17), validation process, and performance estimates.

Table A1

Search Keywords and operationalization in electronic databases

Database	Search entry
PubMed	(machine learning OR machine intelligence OR artificial intelligence OR deep learning OR natural language processing OR machine classifiers OR neural network) AND (cancer OR oncology OR malignancy OR neoplasms) AND (Africa)
EMBASE	Machine learning AND cancer AND Africa
	Machine intelligence AND cancer AND Africa
	Artificial intelligence AND cancer AND Africa
	Deep learning AND cancer AND Africa
	Natural language processing AND cancer AND Africa
	Machine classifiers AND cancer AND Africa
	Neural network AND cancer AND Africa
	Machine learning AND oncology AND Africa
	Machine intelligence AND oncology AND Africa
	Artificial intelligence AND oncology AND Africa
	Deep learning AND oncology AND Africa
	Natural language processing AND oncology AND Africa
	Machine classifiers AND oncology AND Africa
	Neural network AND oncology AND Africa
	Machine learning AND malignancy AND Africa
	Machine intelligence AND malignancy AND Africa
	Artificial intelligence AND malignancy AND Africa
	Deep learning AND malignancy AND Africa
	Natural language processing AND malignancy AND Africa
	Machine classifiers AND malignancy AND Africa
	Neural network AND malignancy AND Africa
	Machine learning AND neoplasms AND Africa
	Machine intelligence AND neoplasms AND Africa
	Artificial intelligence AND neoplasms AND Africa
	Deep learning AND neoplasms AND Africa
	Natural language processing AND neoplasms AND Africa
	Machine classifiers AND neoplasms AND Africa
	Neural network AND neoplasms AND Africa
Web of science	(machine learning OR machine intelligence OR artificial intelligence OR deep learning OR natural language processing OR machine classifiers OR neural network) AND (cancer OR oncology OR malignancy OR neoplasms) AND (Africa)
Cochrane Library	(machine learning OR machine intelligence OR artificial intelligence OR deep learning OR natural language processing OR machine

	classifiers OR neural network) AND (cancer OR oncology OR malignancy OR neoplasms) AND (Africa)
Scopus	Machine learning AND cancer AND Africa
	Machine intelligence AND cancer AND Africa
	Artificial intelligence AND cancer AND Africa
	Deep learning AND cancer AND Africa
	Natural language processing AND cancer AND Africa
	Machine classifiers AND cancer AND Africa
	Neural network AND cancer AND Africa
	Machine learning AND oncology AND Africa
	Machine intelligence AND oncology AND Africa
	Artificial intelligence AND oncology AND Africa
	Deep learning AND oncology AND Africa
	Natural language processing AND oncology AND Africa
	Machine classifiers AND oncology AND Africa
	Neural network AND oncology AND Africa
	Machine learning AND malignancy AND Africa
	Machine intelligence AND malignancy AND Africa
	Artificial intelligence AND malignancy AND Africa
	Deep learning AND malignancy AND Africa
	Natural language processing AND malignancy AND Africa
	Machine classifiers AND malignancy AND Africa
	Neural network AND malignancy AND Africa
	Machine learning AND neoplasms AND Africa
	Machine intelligence AND neoplasms AND Africa
	Artificial intelligence AND neoplasms AND Africa
	Deep learning AND neoplasms AND Africa
	Natural language processing AND neoplasms AND Africa
	Machine classifiers AND neoplasms AND Africa
	Neural network AND neoplasms AND Africa
